# Pure Carbolic in the Treatment of Boils

**Published:** 1908-02-15

**Authors:** J. Lindsay


### The General Practitioner's Column.

[Contributions to this Column are invited, and if accepted will be paid for.]

### PURE CARBOLIC IN THE TREATMENT OF BOILS.

By J. LINDSAY, M.D.

The treatment of the common boil may appear to be a very trivial matter to the medical practitioner, but the small details of the various local measures employed, either in the relief of pain or in cutting short its progress, are matters of no small importance to the unhappy sufferer. It is very often the case that a patient may be afflicted by many crops of boils instead of one boil only; and although he is otherwise healthy, he may be brought to a very low state of health if these recurrences extend over a prolonged period.

In all cases it is good routine treatment to correct errors of diet and constipation, and also to remove any local frictional causes. Constitutional factors, such as diabetes or kidney disease, must always be inquired into as a matter of routine.

The common practice of applying fomentations of carbolic or boric acid is only justified and should only be resorted to when the boil has reached the painful stage—*i.e.* when the tension is great and the inflammation is passing on to suppuration. Here every care should be taken to prevent the pus, when the boil discharges, from infecting the hair follicles in the immediate vicinity, for this will produce a crop of boils, just as occurs experimentally by rubbing a culture of staphylococci over a skin surface.

In applying a fomentation the method which I have found most efficacious is to rub in powdered boric acid over an area of about two inches radius from the seat of infection, then to cut out a square of boracic lint to place over this area, leaving, however, an aperture in the centre of one square inch to allow the boil to remain exposed. The lint is then covered with a layer of oiled silk overlapping all the edges of the lint by a quarter of an inch. Thus the boil will still remain exposed through the aperture of half an inch in the oiled silk. The two layers are held in position by means of strapping, and a fomentation of 1 in 40 carbolic acid is then applied. It should be wrung as dry as possible, and applied as hot as can be borne by the patient; it is then quickly covered with oiled silk, cotton-wool, and a bandage. When the boil bursts, the pus discharges itself through the aperture in the boracic lint into the gauze or lint forming the fomentation, and so cannot spread itself over the adjacent skin surface. Probably the core will not come away for one or two days, in which case the applications will require to be renewed every six hours or so. When the core has separated, the cavity should be carefully wiped out, and the whole exposed area lightly touched up with pure carbolic acid, in order to destroy any remaining staphylococci. An ordinary dressing of double cyanide gauze should then be applied.

A patient who is periodically afflicted with boils knows well when one is coming. Its advent is heralded by symptoms of smarting irritation, with the almost irresistible desire to scratch the part. One of two things may now happen: either in two or three days' time the boil may happily abort; or, more likely, the course will be progressive till the fourth or fifth day, when the tension and pain will be so distressing that any relief in the way of poultices or fomentations is welcomed. It is, however, very desirable that nothing be left to chance, and it is in the early days of its course that the treatment by hypodermic injection of carbolic acid is so uniformly successful, the time of application *par excellence* being at the commencement of the trouble—*i.e.* when the irritation begins.

The exact spot can usually be determined by seeing a minute inflamed papule like a pin-point in the site of a hair follicle, and from which a small hair can be seen projecting. This spot should be touched with the point of a glass rod or blunt end of a needle which has been dipped into pure carbolic, as this renders the spot to some extent anæsthetic. Similarly also a crystal of carbolic acid may be placed over the spot and allowed to liquefy, care being taken to prevent its spreading. Two or three minims of pure carbolic are then drawn into the hypodermic syringe. The needle is entered in a perpendicular direction to a depth of a quarter of an inch, and gradually withdrawn, while the contents of the syringe are steadily injected into the connective tissue underlying the skin.

When pus has formed, the hypodermic injection of carbolic acid is not attended by the same uniform disappearance of the boil. Once this stage has been reached, the pain is so severe as to call for an incision into the boil to relieve tension and create an exit for the pent-up pus and, later on, for the core. Few patients who are the subjects of recurring attacks of boils will submit time after time to the considerable pain attendant on the use of the knife unless nitrous oxide or some other general anæsthetic be administered. Local anæsthetics in such an acute inflammatory condition are of little or no value.

I have recently had the opportunity of carrying out this treatment by applications of pure carbolic in the pustular stage of boils, the idea being to allow the acid to find its way into the boil by destroying the overlying skin and connective tissues. The surrounding area is protected in the manner above described. First one crystal of pure carbolic acid is placed on the apex of the boil, then a second crystal, and so on until the pustule has been opened into. A channel is thus formed to allow of the escape of the pus and central core. A finely pointed probe should then be gently heated and dipped into the bottle containing the crystals of carbolic acid. A few of the crystals will adhere to the probe, the point of which should then be applied over the surface of the small crater—a proceeding which effectually destroys any remaining staphylococci.

This procedure is very effective, the only drawback being the somewhat tedious nature of the process; but it possesses the great advantage of giving no acute pain to the patient, who, at the most, only experiences a slight feeling of discomfort.

